# Non-invasive MRI of choroid plexus vascular function

**DOI:** 10.1162/IMAG.a.1216

**Published:** 2026-04-17

**Authors:** Peiying Liu, Lori Donaldson, Beini Hu, Gagan S. Wig, Hanzhang Lu

**Affiliations:** Department of Diagnostic Radiology & Nuclear Medicine, University of Maryland School of Medicine, Baltimore, MD, United States; Center for Vital Longevity & Department of Psychology, The University of Texas at Dallas, Dallas, TX, United States; Department of Psychiatry, The University of Texas Southwestern Medical Center, Dallas, TX, United States; The Russell H. Morgan Department of Radiology & Radiological Science, Johns Hopkins University School of Medicine, Baltimore, MD, United States

**Keywords:** choroid plexus, vasculature, elasticity, functional MRI, BOLD, aging

## Abstract

Choroid plexus (ChP) is a highly vascularized tissue in the ventricles of the brain, and it plays an important role in the production of cerebrospinal fluid (CSF) and formation of the blood-CSF barrier. The function of ChP vessels has been implicated in waste clearance efficiency during aging and neurodegenerative diseases. At present, postmortem studies are the main method to assess choroid plexus vascular integrity, with a few tools to measure ChP function in living humans. Here, we proposed a non-invasive MRI approach to assess ChP vascular elasticity based on the detection of MRI signal changes in response to vasoactive challenges. The mechanism of the signal is hypothesized to be due to reciprocal blood and stroma volume alterations during vessel expansion. We demonstrated that ChP vascular elasticity can be evaluated with BOLD MRI using a hypercapnia challenge of CO2 inhalation. This effect is specifically located in the brain ventricles where ChP is abundant. We revealed the ability of the technique in detecting age-related reduction in ChP vascular elasticity. We further showed that this effect can be assessed with gas-free methods, including intermittent breath modulation and resting-state BOLD fMRI. We characterized the image contrast requirement under which this effect can be detected. This technique may provide a clinically feasible tool for assessing ChP vascular function in health and disease.

## 1 Introduction

Choroid plexus (ChP) is a highly vascularized secretory tissue found in the lateral, third, and fourth ventricles of the brain, which plays an important role in the production of brain cerebrospinal fluid (CSF) and the formation of the blood-CSF barrier (BCSFB) (Ghersi-Egea et al., 2018). The significance of ChP in brain health and disease is underscored by its involvement in waste clearance (Spector et al., 2015) and as part of a possible glymphatic system of the brain (Lehtinen et al., 2013). CSF circulation has also been hypothesized to play a central role in several proteinopathy-centric diseases, including Alzheimer’s disease (AD), Parkinson’s disease (PD), and Huntington’s disease (HD), because impaired clearance of toxic proteins leads to neurodegeneration and cognitive decline (Iliff et al., 2012). However, there have been few tools available to assess vascular function of ChP in living humans. Recently, arterial spin labeling (ASL) perfusion MRI has been suggested to be a potential non-invasive tool for the quantification of ChP perfusion (Eisma et al., 2023; Evans et al., 2020; Lee et al., 2022; Zhao et al., 2020). A few studies have reported a decrease in ChP blood flow with aging in human (Alisch et al., 2021; Sun, Li, Zhang, et al., 2024). However, another important aspect of the vascular function of ChP vessels, their vascular elasticity, remains unclear due to a lack of feasible tools.

**Fig. 1.**
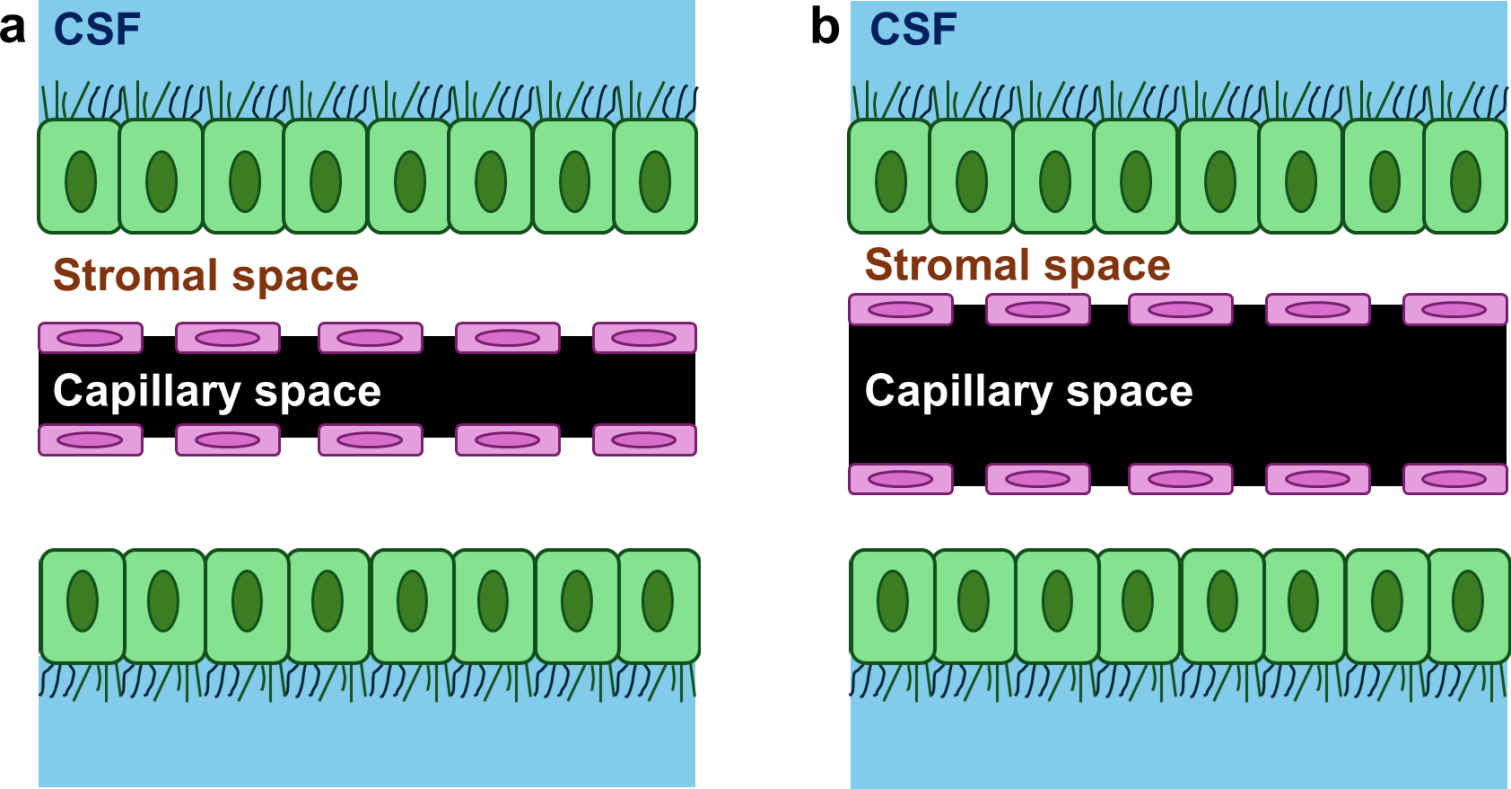
Illustration of the components in an imaging voxel of the ChP. (a) Capillary and stroma share the space inside the ChP epithelial cells during baseline conditions. (b) capillary space increases and stoma space decreases when the ChP capillary diameter increases during vasoactive challenges.

BOLD MRI in response to vasoactive challenges has been used to evaluate vascular function in the brain parenchyma, often referred to as cerebrovascular reactivity (CVR). However, MRI signals in the ChP have not been fully characterized, although they have been occasionally discussed in the context of CVR artifacts (Thomas et al., 2013). The plexuses in ChP comprised fenestrated vessels, a single layer of choroid epithelial cells, and a stroma of fluid and connective tissue between them (Ghersi-Egea et al., 2018). We hypothesize that the MRI signal will manifest an alteration due to reciprocal blood and stroma volume changes during vasoactive challenges. As shown in Figure 1a, fenestrated capillaries and stroma occupy the space within the enclosure of the epithelial cells. When perfusion pressure increases such as during vasodilatory stimulation by hypercapnia, the ChP vessel diameter will increase despite the absence of smooth muscle cells. The increase in capillary space will lead to a decrease in stroma space, as illustrated schematically in Figure 1b. Importantly, blood and stroma have different MR properties. It is expected that the stromal space has MR properties similar to that of plasma. This is because the stroma space is immediately adjacent to the ChP vessels and these vessel walls are leaky. Thus, other than red blood cells, the contents of the stroma are similar to that of the blood (Lun et al., 2015). Plasma is known to have a substantially longer T1 and T2 compared to the blood (Li et al., 2016; Li & van Zijl, 2020). As a result, a change in blood/stroma space occupancy, that is, a partial volume effect, will lead to an alteration in MRI signal in the imaging voxels containing ChP. To differentiate this ChP space occupancy change from oxygenation change that typically results in a signal increase, we will focus on MRI pulse sequences that result in fluid signal being brighter than blood and tissue. With this image contrast, ChP vessel expansion will result in a signal decrease, which is characteristically different from the oxygenation-related signal increase.

In this report, we performed three studies to develop the methods and test the hypothesis. We first tested this technique using a hypercapnia challenge in 192 healthy subjects with varying ages across the adult lifespan. The hypercapnia challenge used 5% CO2 inhalation. We evaluated the feasibility of measuring MRI signal decreases associated with ChP vascular elasticity and investigated its differences in aging. We then investigated if ChP vascular elasticity can be assessed without hypercapnia challenges, as administration of the hypercapnia challenges requires the access of the gas delivery system which limits its application in standard clinical settings. We used a gas-free approach of breath modulation in a group of healthy younger and older subjects, and examined if the MRI signal decreases associated with ChP vascular elasticity can be observed. Finally, since resting-state fMRI is the most widely applied BOLD acquisition, we further determined the pulse sequence conditions under which ChP vascular elasticity can be measured using resting-state MRI without any overt tasks or challenges.

## 2 Methods

### 2.1 Study 1: Choroid plexus vascular elasticity measured by hypercapnia challenge

We assessed ChP vascular elasticity using hypercapnia MRI in a group of 192 healthy subjects between 20 to 88 years of age. These subjects were part of the cohort of the prospective, longitudinal Dallas Lifespan Brain Study (Park et al., 2025). This study was approved by the Institutional Review Board of the University of Texas Southwestern Medical Center and the University of Texas at Dallas. All participants underwent extensive health screening and had no contraindications to MRI scanning (pacemaker, implanted metallic objects, claustrophobia) and were generally of good health, with no serious or unstable medical conditions such as neurological disease, brain injury, uncontrollable shaking, history of bypass surgery or chemotherapy, or use of medications that affect cognitive function. All participants were highly right-handed, native English speakers with at least a high school education, and a Mini-Mental State Exam (MMSE) (Folstein et al., 1975) score of 26 or greater. Each subject gave informed written consent before participating in the study.

All MR imaging experiments were conducted on 3 Tesla MR system (Philips Medical System, Best, The Netherlands). Each subject underwent a mild hypercapnia challenge while blood oxygen level–dependent (BOLD) MRI images were acquired continuously. Specifically, subjects were fitted with a mouthpiece and a nose clip, and CO2-enriched air (5% CO2, 21% O2, and 74% N2) was administered with a Douglas airbag, with a valve to switch between room air and CO2-rich air. Subjects breathed room-air and 5% CO2 in an interleaved fashion (switching every 1 min) for a total of 7 min. End-tidal CO2 (EtCO2) was recorded continuously during the scan using a capnograph device (Capnogard, Model 1265, Novametrix Medical Systems, CT). BOLD MRIs were continuously acquired during the entire 7 min period using the following scan parameters: gradient echo planar imaging (EPI), TR/TE/flip angle = 2000 ms/25 ms/80°, field-of-view (FOV) = 220 × 220 mm^2^, matrix size = 64 × 64, voxel size = 3.4 × 3.4 × 3.5 mm^3^, and 43 axial slices with whole-brain coverage. In addition, a T1 magnetization-prepared rapid acquisition gradient echo (MPRAGE, voxel size 1 × 1 × 1 mm^3^, duration 4 min) was acquired for anatomic reference.

The data analysis was conducted using the software Statistical Parametric Mapping (SPM12) (University College London, UK) and in-house MATLAB scripts. After motion correction and spatial smoothing (by a Gaussian FWHM kernel of 4 mm), a whole-brain averaged BOLD signal time course was calculated and used to obtain the synchronized EtCO2 time course. The synchronization between the Et-CO2 time course and the BOLD time course was achieved by introducing a delay to the recorded Et-CO2 time course, at which the Et-CO2 yielded the lowest sum-of-square residual with the BOLD time course. Next, voxel-wise general linear model (GLM) analysis was performed in which the BOLD signal intensity time course was the dependent variable and synchronized EtCO2 time course was the independent variable, with a linear trend as a covariate (Lu et al., 2014). The coefficients of the regression analysis yielded a voxel-wise map in the units of %ΔBOLD per mmHg of CO2. Finally, the individual maps were normalized into the Montreal Neurological Institute (MNI) template space via T1-MPRAGE for group-level statistical analyses. These analysis steps are similar to those employed in CVR MRI data. However, we emphasize that, in this study, we are primarily interested in signal *decrease* with CO2 inhalation, as opposed to signal *increase* as in CVR assessment.

In addition, CSF signal intensities in the lateral ventricles and tissue signal surrounding the lateral ventricles were evaluated using SPM segmentation of T1-MPRAGE which was mapped to the mean BOLD image to calculate a CSF/tissue contrast ratio for each subject.

Group-level voxel-wise analyses were performed using SPM12. Specifically, detection of significant voxels with negative CO2 responses was performed using a one-sample t-test comparing each voxel of all subjects to 0, with a threshold of p < 0.005 and cluster size > 50 voxels. Detection of significant voxels with age-related increase was performed using a regression analysis with age as the regressor. Voxels with a significant age-increase were identified with a threshold of p < 0.005 and cluster size > 50 voxels.

ROI analysis based on the significant clusters with negative CO2 responses was also performed. The relationship between ROI CVR value and age was evaluated by linear regression analysis with age of each individual as independent variable and the individual ROI CVR value as dependent variable. Sex was included as a covariate. A p value <0.05 is considered statistically significant.

### 2.2 Study 2: Choroid plexus vascular elasticity measured by intermittent breath modulation

We investigated whether ChP elasticity can be examined with a gas-free approach referred to as intermittent breath modulation (Liu et al., 2020). In this study, we recruited 27 healthy adult subjects, including 17 younger subjects (age 26.6 ± 4.8 years, 9 males, 8 females) and 10 older subjects (age 60.1 ± 6.6 years, 6 males, 4 females). This study was approved by the Institutional Review Board of Johns Hopkins University and the University of Maryland School of Medicine. The subjects had no contraindications to MRI scanning and were generally of good health, with no current medical conditions or previous history of neurological diseases, psychiatric diseases, brain trauma, diabetes, cardiovascular and cerebrovascular diseases, pulmonary diseases, or use of medications that affect cognitive function. All subjects had a Montreal Cognitive Assessment (MoCA) (Nasreddine et al., 2005) score of 26 or higher, suggesting normal cognitive function in these subjects.

All MR imaging experiments were conducted on 3 Tesla Siemens MR system (Siemens Healthineers, Erlangen, Germany). Each subject performed an intermittent breath modulation task (Liu et al., 2020) for 7 min. The task consisted of 7 paced-breathing periods, each including 2 s breathe-in/2 s breathe-out for three times, and interleaved with free-breathing periods. The durations of the free-breathing periods were randomized to be between 30-60 s, to avoid any anticipation effects on the participant. Our previous work showed that such an intermittent breath modulation paradigm can effectively introduce variations in the subject’s end-tidal CO2 during the BOLD MRI run (Liu et al., 2020). During the task, BOLD MR images were continuously acquired using the following imaging parameters: gradient EPI, TR/TE/flip angle = 1500 ms/37 ms/52°, field-of-view (FOV) = 208 × 208 mm^2^, matrix size = 104 × 104, voxel size = 2 × 2 × 2 mm^3^, multiband factor = 8, and 72 axial slices with whole-brain coverage. A T1-MPRAGE scan (voxel size 1 × 1 × 1 mm^3^) was also acquired for anatomic reference.

The data processing followed steps similar to those in Study 1. The only difference is that, since there was no CO2 inhalation in this study, EtCO2 was not used as a regressor. Instead, the regressor used the whole-brain averaged BOLD signal time course, after detrending and filtering with a low-pass Butterworth filter with a cutoff of 0.1164 Hz (Liu et al., 2021). The voxel-wise BOLD signal time course was used as the dependent variable. The motion vectors and a linear trend were included as co-variates. The voxel-wise coefficient from the regression was then normalized by their whole-brain mean value to yield a relative map. Finally, the individual relative maps were transformed into MNI template space via T1-MPRAGE for group-level statistical analyses to detect negative coefficient values.

Voxel-wise analysis similar to that used in Study 1 was employed to identify voxels with a negative response. For ROI analysis, the younger and older subjects were separately analyzed. Group differences in ROI CVR values were evaluated by linear regression analysis with group of each individual as independent variable, individual ROI CVR value as dependent variable, and sex as a covariate.

### 2.3 Study 3: ChP vascular elasticity assessment with resting-state fMRI

Since resting-state fMRI is the most widely applied BOLD acquisitions, we further investigated whether ChP vascular elasticity can also be assessed using a resting-state BOLD fMRI scan. In this study, 89 de-identified multi-echo datasets were downloaded from the OpenfMRI database (https://openfmri.org/dataset/ds000258/). These multi-echo datasets were previously reported in a study by Power et al. (2018), and collected from normal adults from whom informed consent was obtained in a study approved by the Local Research Ethical Committee at the University of Cambridge. These BOLD datasets were collected during a 9.8 min resting-state scan on a 3 Tesla Siemens MRI scanner using the following imaging parameters: multi-echo EPI, TR = 2470 ms, four echoes with TE = 12, 28, 44, and 60 ms, flip angle = 78°, field-of-view (FOV) = 240 × 240 mm^2^, matrix size = 64 × 64, in-plane resolution = 3.75 × 3.75 mm^2^, 32 oblique slices with slice thickness of 3.75 mm, and 10% gap. A T1-MPRAGE dataset (voxel size 1 × 1 × 1 mm^3^) from each subject was also downloaded. One dataset was discarded due to corrupted BOLD data of echo 2.

For each of these four-echo datasets, BOLD images from each echo were processed separately following the imaging processing steps described in Study 2, yielding four relative CVR maps corresponding to each echo. The CSF/tissue contrast ratio was also calculated for each echo of each subject as described above.

Group-level voxel-wise analysis was performed to identify negative-response voxels in each echo. ROI CVR values and CSF/tissue contrast ratio obtained from each echo were compared to 0 using one-sample t-test.

## 3 Results

### 3.1 Choroid plexus vascular elasticity measured by hypercapnia challenge

**Fig. 2.**
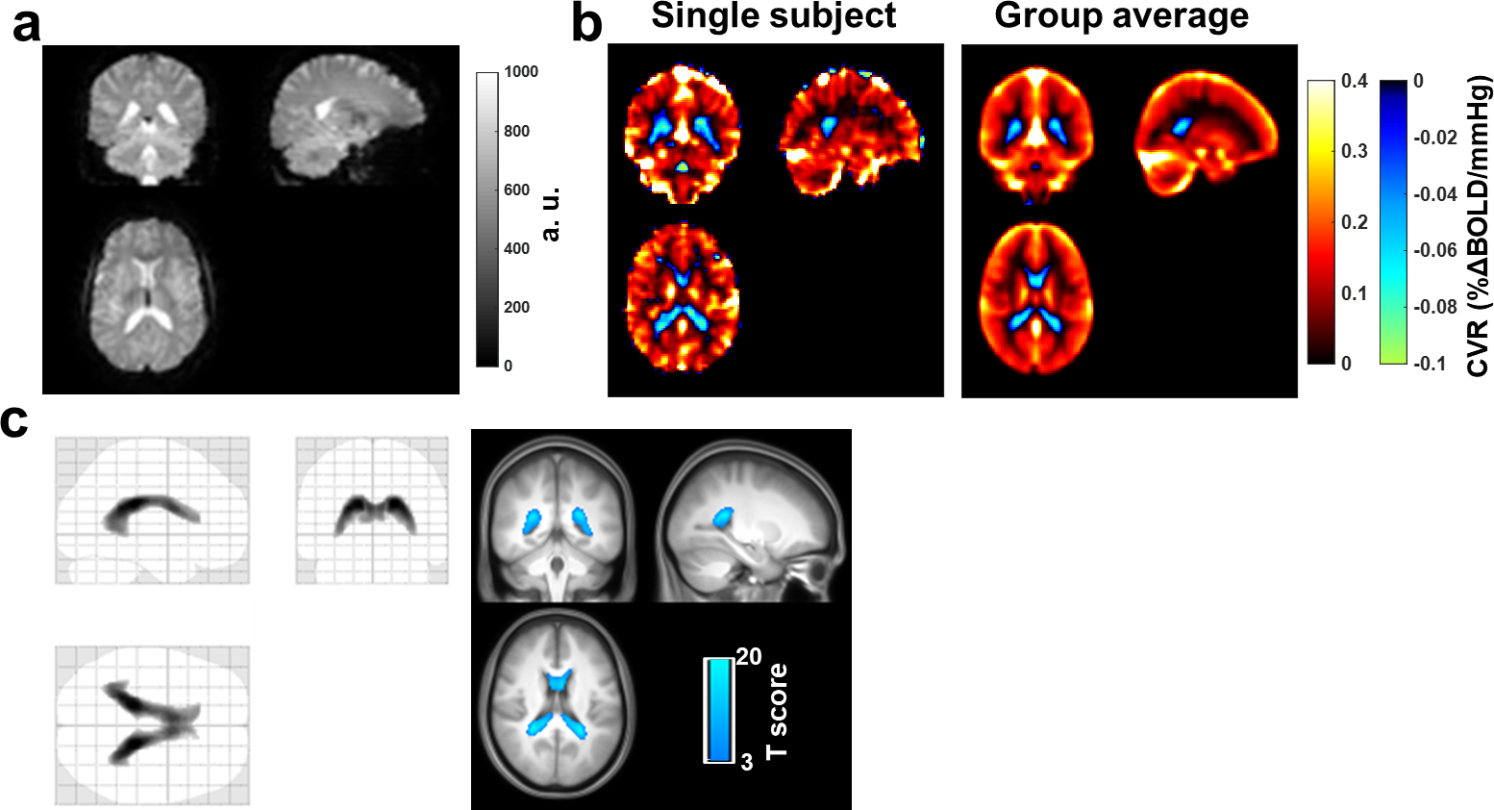
Negative CVR in lateral ventricles represents ChP vascular elasticity. (a) Representative BOLD image from one subject. Note that ventricles are considerably brighter than brain parenchyma. (b) CVR maps (in %ΔBOLD/mmHg CO2) measured with hypecapnia challenge from a 56-year old subject (left) and averaged across all 192 subjects (right). The CVR maps were displayed at the conventional positive range for gray and white matter. Regions with negative CO2-CVR values are illustrated with cool color. (c) Results of voxel-based one-sample t-test analysis of negative CVR. The left figure shows the glass brain overlay, and the right figure shows the overlay of the thresholded spm T map on group-averaged MPRAGE image. Colored voxels indicate voxels with significant negative CO2-CVR (p < 0.005, cluster size>50 voxels).

In Study 1, the BOLD MRI sequence used (relatively long TR and TE of 2000 and 25 ms, respectively) allowed the CSF and stroma fluid signal to be hyperintense relative to the tissue and blood signal (Fig. 2a). The mean CSF/tissue contrast ratio was 1.44 ± 0.12 across all subjects, and was not associated with age (p = 0.70). This is critical to allow the sequence to differentiate the effects of blood volume increase in ChP from the effects of blood flow or oxygenation increase. Specifically, blood volume effects will result in a signal decrease while blood flow and oxygen effects will result in an increase.

As shown in Figure 2b, reliable CVR maps can be obtained from individual subjects and averaged across the entire cohort. Notably, negative CVR values can be seen in the lateral ventricle area where ChP is known to present. Group-level voxels with statistically significant negative CVR values are shown in Figure 2c. The presence of negative CVR voxels in the lateral ventricles supports our hypothesis that dilation of capillaries in ChP during CO2 inhalation causes the volume decrease in stroma, leading to a BOLD signal reduction.

### 3.2 Age-related changes in ChP vascular elasticity measured by hypercapnia challenge

**Fig. 3.**
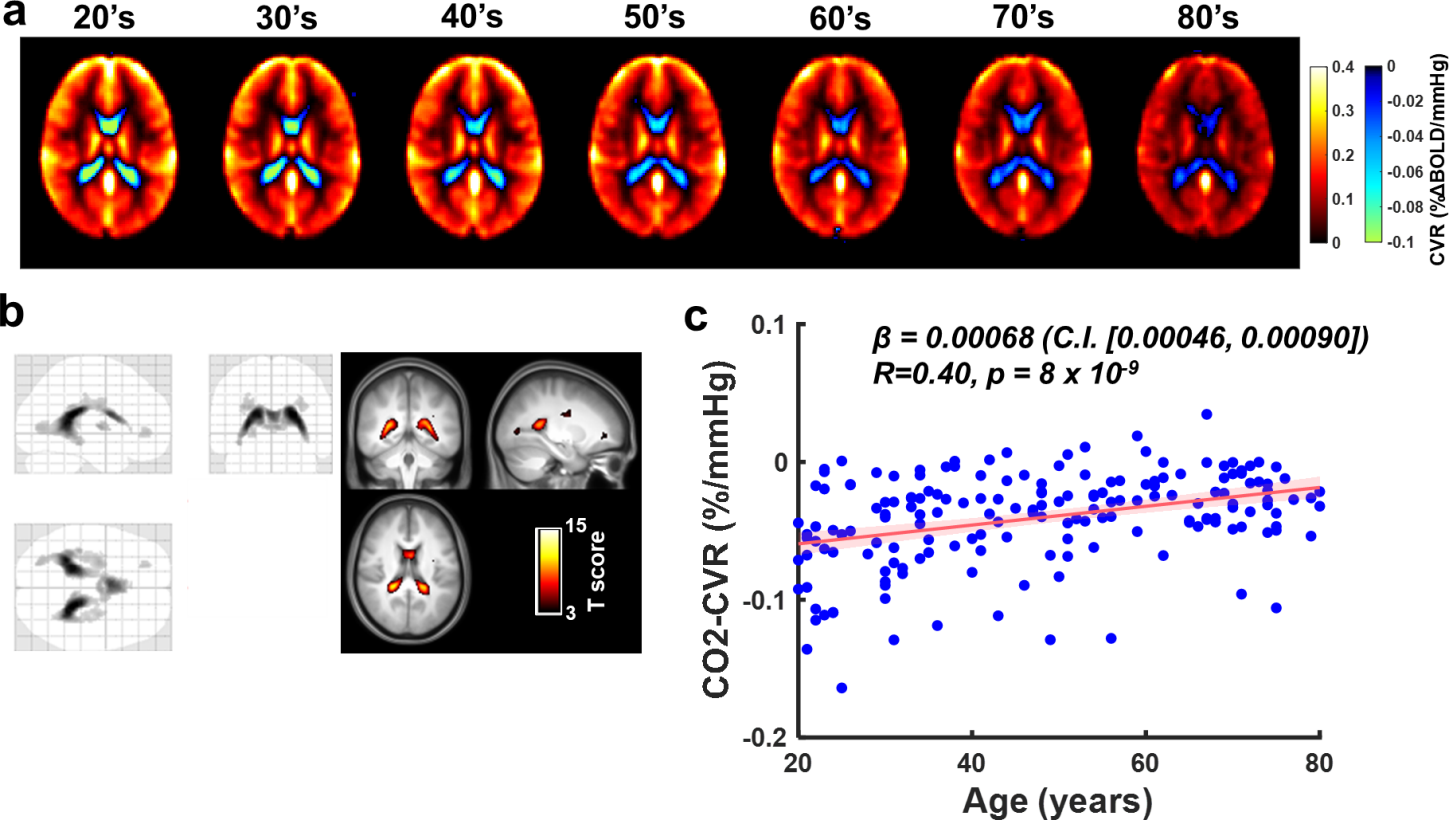
Age-related differences of ChP vascular elasticity measured by negative CVR in lateral ventricles. (a) Decade-by-decade results of cerebrovascular reactivity (CVR). Negative CVR values are illustrated by cool colors. (b) Results of voxel-based regression analysis of age-related increases in CO2-CVR values. The left figure shows the glass brain overlay, and the right figure shows the overlay of the thresholded spm T map on group-averaged MPRAGE image. Colored voxels indicate voxels with significant age-related increase (p < 0.005, cluster size>50 voxels). Note that the apparent age-related increase in ventricle voxels with negative CVR values indicates age-related dampening of ChP vascular elasticity. (c) Scatter plot between age and CO2-CVR in voxels showing negative CVR (i.e., voxels identified in Fig. 2c). Each dot represents one subject. Red line indicates linear regression. The shaded areas indicate the 95% confidence interval (C.I.).

Decade-by-decade averaged CVR maps from Study 1 are shown in Figure 3a. Visual inspection revealed that gray matter CVR decreased with age, consistent with previous literature. In the ventricular regions, fewer negative CVR voxels with smaller negative values were observed in older age individuals. Voxel-wise results of age-related changes are shown in Figure 3b. It can be seen that the lateral ventricles revealed an age-related increase, mainly in the area where the negative CVR voxels are present, due to age-accompanied attenuation of negative CVR values in this area. Using the voxels with significant negative CVR values from the group analysis shown in Figure 2c as the region-of-interest, ROI-averaged CVR values were calculated for each subject. CVR values changes from -0.061 ± 0.041 %/mmHg in subjects in their 20 s to -0.018 ± 0.021 %/mmHg in subjects in their 80 s. As shown in Figure 3c, regression analysis revealed a significant positive association (β = 0.00068, C.I. [0.00046, 0.00090], p = 8.1 x 10^-9^) between CVR and age across all subjects, where the negative CVR values decreased (i.e., less negative) as age increased. Similar observations were found when using voxels delineated in Figure 3b. The age-related decrease in negative CVR suggests dampened vascular elasticity in ChP among older age individuals.

### 3.3 Gas-free assessment of ChP vascular elasticity assessment

**Fig. 4.**
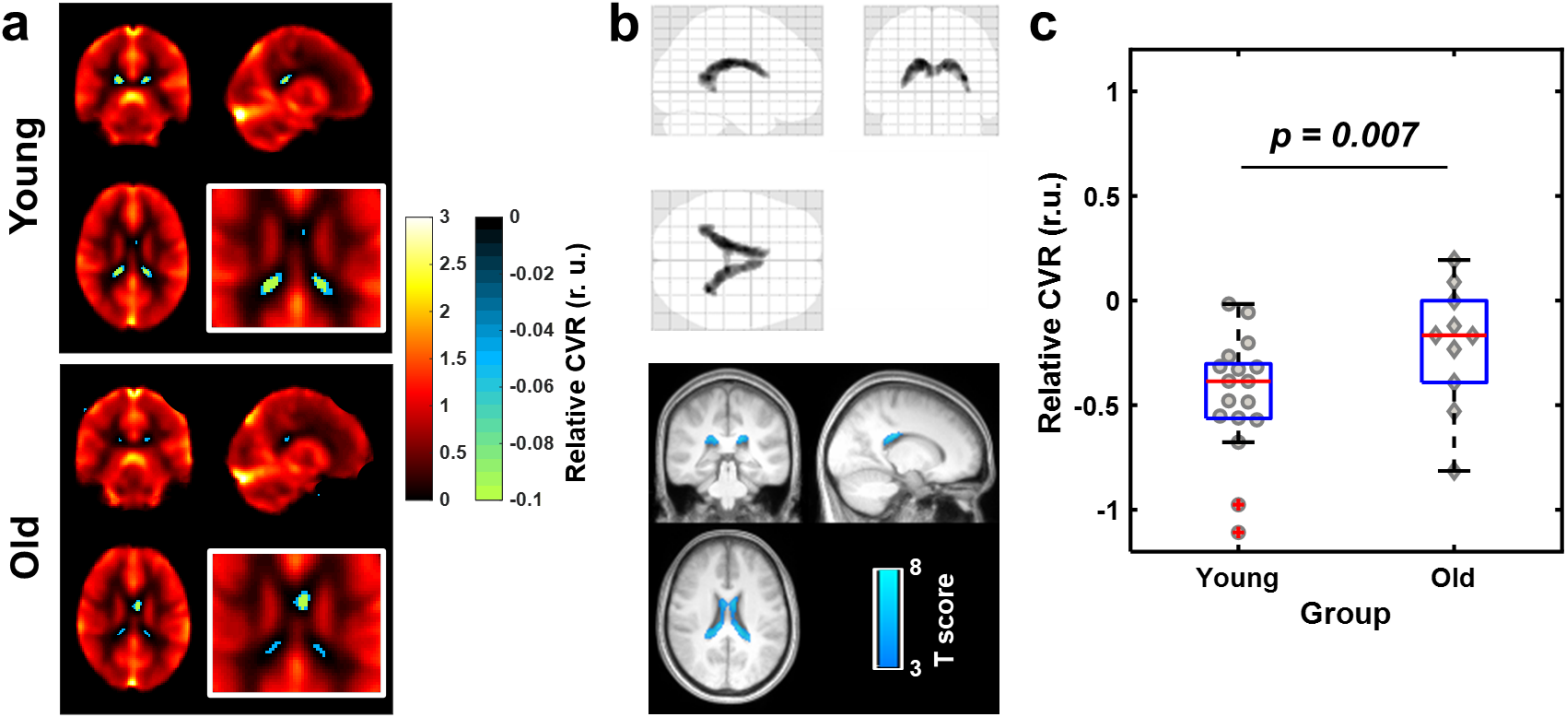
ChP vascular elasticity was assessed by negative CVR using intermittent breath modulation. (a) Group-averaged relative CVR maps (in relative unit (r.u.)) from 17 younger subjects (top) and 10 older subjects (bottom), respectively. Negative CVR values are illustrated by cool color). Zoomed-in views of the ventricular region are shown in the white boxed insets. (b) Results of voxel-based analysis of negative CVR in all 27 subjects. The top figure shows the glass brain overlay, and the bottom figure shows the overlay on group-averaged MPRAGE image. Colored voxels indicate voxels with significant negative relative CVR (p < 0.005, cluster size>50 voxels). (c) Box plot of negative CVR values compared between the young and old groups. Gray symbols represent individual subjects. Central bars and bottom and top edges of the box indicate the medians and 25th and 75th percentiles, respectively. The whiskers indicate the minimum and maximum, excluding outliers. Red crosses indicate outliers.

Figure 4a shows the averaged relative CVR maps obtained using intermittent breath modulation for the young and old groups, respectively. Voxels with negative CVR values were present in the ventricles in both groups. Group analysis of all 27 subjects together confirmed that voxels with significant negative CVR values were located within the lateral ventricles, as shown in Figure 4b. ROI analysis was also performed using the voxels showing negative CVR in Figure 4b as identified from this group analysis. The results are shown in Figure 4c. The old group showed significantly higher relative CVR (smaller negative values) than the young group in the ROI (-0.46 ± 0.26 r.u. and -0.15 ± 0.26 r.u. for young and old, respectively; p = 0.007). These results of Study 2 are consistent with our findings in Study 1 using hypercapnia challenges, suggesting that ChP vascular elasticity can also be assessed with a gas-free approach.

### 3.4 ChP vascular elasticity assessment with resting-state fMRI

**Fig. 5.**
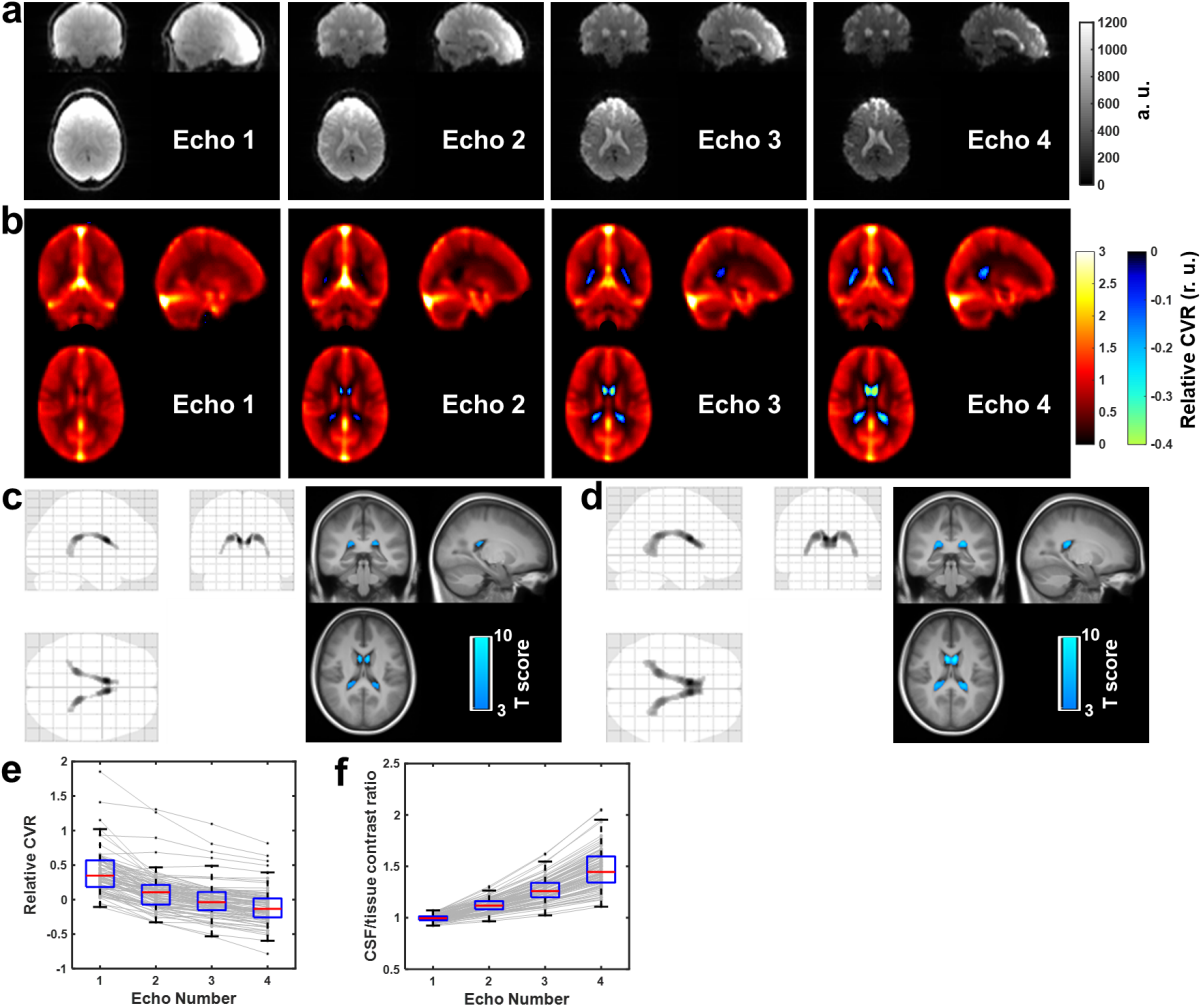
ChP vascular elasticity was assessed by negative CVR multi-echo (ME) resting state BOLD fMRI. (a) Raw BOLD images obtained from each echo of the ME scan of a representative subject. (b) Group-averaged relative CVR maps (in relative unit (r.u.)) across 88 healthy participants. Cool color illustrates voxels with negative values. Voxel-based analysis identified significant voxels of negative CVR in (c) Echo 3 and (d) Echo 4 in all 88 subjects. The left figure shows the glass brain overlay, and the bottom figure shows the overlay on the group-averaged MPRAGE image. Colored voxels indicate voxels with significant negative relative CVR (p < 0.005, cluster size > 50 voxels). Box plots of (e) negative CVR values within the ROI determined from Echo 4 as shown in (d), and (f) CSF/tissue signal contrast ratio as a function of TE (i.e., different echos) are also shown. Gray dots represent individual subjects and the same subjects were indicated by connected gray bars. Central bars (red lines) and bottom and top edges of the box indicate the medians and 25th and 75th percentiles, respectively. The whiskers indicate the minimum and maximum, excluding outliers.

Figure 5a shows the raw BOLD images from each echo for a representative subject in Study 3. It can be seen that as the TE became longer, the contrast between CSF and surrounding brain tissue was larger. Figure 5b shows the group-averaged relative CVR maps obtained from each echo. As the TE increases, voxels with negative CVR became more apparent. Group analysis confirmed that significantly negative CVR voxels were present in Echo 3 (TE = 44 ms, Fig. 5c) and Echo 4 (TE = 60 ms, Fig. 5d) where larger CSF/tissue contrast is shown in raw BOLD images among the four echoes. These negative voxels were located within the lateral ventricles. Figure 5e displays the CVR values in lateral ventricles as a function of TE, and Figure 5f illustrates CSF and tissue contrast as a function of TE. These results indicate that ChP vascular elasticity can also be assessed by resting-state BOLD fMRI, but only with imaging parameters that yield large CSF/tissue contrast.

## 4 Discussion

The physiological integrity of vessels inside the ChP plays a critical role in maintaining its normal function, such as CSF production, waste clearance, and formation of BCSFB. However, there has been a scarcity of studies investigating vascular function of ChP, due to a lack of suitable techniques to measure these cells. In this work, we proposed a non-invasive MRI approach to assess the elasticity of blood vessels in ChP. We demonstrated that ChP vascular elasticity can be evaluated with BOLD MRI using a hypercapnia challenge with CO2 inhalation. We revealed the ability of the proposed technique in detecting age-related differences in ChP vascular elasticity. We further showed that this effect can also be assessed with gas-free methods, including intermittent breath modulation and resting-state BOLD fMRI. Moreover, we conducted technical characterization of the signal and revealed that a pre-requisite to measure the effect is that the CSF region in the BOLD image needs to be brighter than the tissue. This condition ensures that the pulse sequence is sensitive to the blood volume effect.

Choroid plexus (ChP) has received increasing attention due to its role in normal aging and neurodegenerative diseases. Decreased CSF production, compromised BCSFB integrity, and reduced waste clearance have been postulated to be an intricate step in brain pathogenesis, but such hypotheses have been insufficiently tested in humans especially under *in vivo* conditions. As one of its key components, blood vessels in ChP undergo various changes in health aging and age-related disease, such as reduced vascular area, increased amyloid and other protein deposition in the vessel walls, worsened calcification, thickening of vessel walls, and basement membrane (Masseguin et al., 2005; Samardzija et al., 2021). These changes in vascular ultrastructure cannot yet be visualized using modern medical imaging, but can be assessed with functional imaging tools such as those proposed in the present study. Thickening of vessel walls and basement membrane is expected to lead to more rigid ChP vessels in the aging brains, which is consistent with our finding of age-related reduction in ChP vascular elasticity. The vascular elasticity of ChP is essential for providing a driving force to support CSF production (Lun et al., 2015). Reduced ChP vascular function can lead to impaired plasma filtration, reduced CSF turnover, and diminished clearance of metabolites. These changes can further impair waste processing and clearance in the brain (Sun et al., 2025; Tsitsou-Kampeli et al., 2024). Therefore, the proposed technique has the potential to open new avenues in better understanding the role of ChP function in aging and neurological diseases.

To our knowledge, the present study is the first report on the measurement of the elasticity of ChP vessels. A few prior studies reported a decrease in ChP blood flow with aging in animals and human subjects. Studies using diffusion MRI reported more unrestricted movement of water in ChP in the elderly than in the young (Alicioglu et al., 2017; Alisch et al., 2021; Sun, Li, Zhang, et al., 2024). These findings are thought to be associated with age-decrease in vessel density in ChP (Sun, Li, Muccio, et al., 2024). On the other hand, our technique provides insights on the dynamic functional properties of ChP vessels and evaluates the elasticity of ChP vessels, beyond the structure or density assessment.

It is worthwhile to discuss the signal mechanism of the proposed ChP elasticity technique. BOLD MRI studies via the modulation of arterial CO2 content have primarily been used to investigate cerebrovascular reactivity (CVR) (Blockley et al., 2017; Bright & Murphy, 2013; Liu et al., 2019). However, it is important to point out that the signal reported in the present study has a categorically different mechanism. Our study focused on a negative BOLD signal change, while the CVR studies have typically focused on positive signal changes. A negative CVR has been previously reported, some attributing it to a stealing effect (Conklin et al., 2010; Ishii et al., 2020; Poublanc et al., 2013) and others considering it as an artifact to remove (Thomas et al., 2013). This present study is the first to exploit this negative signal and its spatial localization as a new contrast indexing the ChP vessel function. While the BOLD signal measured in CVR assessment is based on oxygenation-related T2* effects, the signal measured in this study is due to changes in partial volume between different tissue types. Our interpretation is that the observed signal decrease is due to a partial volume reduction in the ChP stroma space secondary to an expansion of blood volume. While we are unable to measure the stroma MR signal intensity directly due to the tiny space it occupies, we estimate that the stroma T2 is substantially longer than the blood T2 given the absence of blood cells in the stroma (Lun et al., 2015). Consistent with this expectation, a previous study reported that plasma T2 at 3T is 410 ms, which is considerably longer than the typical blood T2 of ~100 ms (Li & van Zijl, 2020). Thus, the stroma signal is greater than the blood signal under the BOLD sequence employed in our acquisition. When the volume fraction of the high-intensity compartment is reduced, an overall signal decrease is expected. This effect is conceptually similar to the blood-volume-based VASO-fMRI signal (Lu et al., 2003) that is well-known in the fMRI field. Another possible reason of the observed negative BOLD signal could be a blood oxygenation change in the ChP vessels. The oxygen metabolic rate in the ChP tissue and epithelial cells are thought to be low, thus the blood in the ChP is expected to have a higher oxygenation level compared to the brain parenchyma. Therefore, during hypercapnia, the blood may be redirected to metabolically more demanding regions, resulting in a reduced blood flow and oxygenation. While this is a possible explanation, a previous study using FLAIR-like sequence to make CSF darker has successfully showed that the negative signal became positive, suggesting that it was not due to the BOLD effect (Thomas et al., 2013). In the present study, we investigated the TE-dependence of the signal (in Study 3) and observed that the signal flipped its sign when going from a shorter to a longer TE, which is not expected for a T2* effect. Therefore, our data did not suggest oxygenation change as a major contributor of the observed signal.

We attributed the blood-volume increase in the ChP primarily to a passive expansion of endothelial cells forming the blood vessels. Unlike the case in the cerebral cortex, ChP fenestrated capillaries do not contain smooth muscle cells. Although the endothelial cells of the fenestrated capillaries in ChP are wrapped by pericytes (Lun et al., 2015), the role of pericytes in active vasodilation remains controversial (Dalkara et al., 2024; Fernández-Klett et al., 2010; Hill et al., 2015). Since fenestrated capillaries constitute the majority of ChP vessels, we speculate that the passive expansion of the vessel is due to an elevation in intramural pressure during hypercapnia, which increases the vessel lumen similar to the case of inflating a balloon. Thus, the extent of the expansion, that is, the magnitude of the negative MRI signal, primarily reflects the elasticity of the vessel wall and the thickness of the surrounding basement membrane. On the other hand, we cannot rule out the possibility that a small fraction of the observed signal is attributed to active dilation of small arteries and arterioles supplying the ChP. These vessels possess smooth muscle cells and are surrounded by bright CSF fluid. Therefore, when they actively dilate, the CSF partial-volume is reduced, resulting in a signal decrease. Based on the spatial location and scope of the negative signal changes observed in our studies, we do not anticipate this to be a major component of our signal source. However, further studies, for example, high-resolution functional imaging combined with angiogram to visualize the choroidal arteries, are needed to better quantify the relative contributions of passive and active vasodilation to the observed signal.

This study revealed, for the first time, that negative BOLD MRI signal change can not only be observed during CO2 inhalation, but also during breath-modulation and resting-state scans. This finding has significant practical implications. CO2 inhalation inside the MRI scanner is known to be a complex procedure and may present scalability challenges when applied to multi-site trials or routine clinical MR imaging. Our demonstration of measuring ChP vascular elasticity using resting-state fMRI laid a foundation to evaluate ChP vascular function in broader clinical applications. It also enables retrospective analysis using existing resting-state data available in the literature that contains suitable imaging parameters (e.g., single-echo data with sufficiently long TE or multi-echo data). A caveat that should be noted is that not all resting-state datasets can be used to assess ChP vascular elasticity. Our results suggested that CSF/tissue contrast can serve as an empirical indication in determining the sensitivity of the BOLD MRI signal to ChP elasticity. If the BOLD sequence used a short TE and/or short TR, the CSF signal will be dampened relative to the tissue. Such data will not be able to be used for ChP assessment.

This study is a proof-of-principle and demonstrates the feasibility of measuring ChP vascular elasticity non-invasively in human subjects. Future studies are needed to further develop the technique and identify the optimal imaging parameters for robust measurement of ChP vascular elasticity, especially for the resting-state scans. Besides TR and TE, voxel size may also affect the sensitivity in detecting ChP elasticity. Despite the variety of imaging resolutions used in the three studies, voxel size should also be optimized in future studies. Additionally, in the present study, the index obtained was based on BOLD signal percentage change but does not have a physiological meaning. Future work should develop biophysical models and improved data acquisitions to derive a physiologically meaningful parameter such as blood volume change. In particular, partial voluming from the ventricular CSF does not affect the absolute signal change observed, but could influence the baseline signal thereby altering the percentage signal change. A simple calculation indicated that, the more the voxel contains ventricular CSF, the smaller the percentage change will be. Another limitation is that we only focused on ChP in the lateral ventricles. We did not assess ChP elasticity in the third, fourth ventricles, or cerebral aqueduct due to smaller volumes of these structures. Further development of imaging technologies may allow the assessment of these structures.

In conclusion, we have proposed a non-invasive MRI technique to evaluate ChP vascular elasticity in humans and reported the first observation of age-related reduction in vascular elasticity in ChP. This technique may provide a clinically feasible tool for assessing ChP vascular function in health and disease across the human lifespan.

## Data Availability

All data of Study 1 from the DLBS are available open access on OpenNeuro.org (https://openneuro.org/datasets/ds004856). All data of Study 2 are available open access on OpenNeuro.org (https://openneuro.org/datasets/ds007588). All data of Study 3 were downloaded from OpenfMRI (https://openfmri.org/dataset/ds000258/). The codes used in this work are available at https://github.com/BrainPhysioMRI/ChP_elasticity
